# Effects of metastasis-associated in colon cancer 1 inhibition by small hairpin RNA on ovarian carcinoma OVCAR-3 cells

**DOI:** 10.1186/1756-9966-30-83

**Published:** 2011-09-16

**Authors:** Ruitao Zhang, Huirong Shi, Zhimin Chen, Qinghua Wu, Fang Ren, Haoliang Huang

**Affiliations:** 1 Department of Obstetrics and Gynecology, First Affiliated Hospital, Zhengzhou University, NO.1 Jianshe Road, Zhengzhou, Henan, 450052, P.R. China

**Keywords:** Ovarian carcinoma OVCAR-3 cells, Metastasis-associated in colon cancer 1, Small hairpin RNA, Therapy target

## Abstract

**Background:**

Metastasis-associated in colon cancer 1 (MACC1) is demonstrated to be up-regulated in several types of cancer, and can serve as biomarker for cancer invasion and metastasis. To investigate the relations between MACC1 and biological processes of ovarian cancer, MACC1 specific small hairpin RNA (shRNA) expression plasmids were used to investigate the effects of MACC1 inhibition on ovarian carcinoma OVCAR-3 cells.

**Methods:**

Expressions of MACC1 were detected in different ovarian tissues by immunohistochemistry. MACC1 specific shRNA expression plasmids were constructed and transfected into OVCAR-3 cells. Then, expressions of MACC1 were examined by reverse transcription polymerase chain reaction (RT-PCR) and Western blot. Cell proliferation was observed by MTT and monoplast colony formation assay. Flow cytometry and TUNEL assay were used to measure cell apoptosis. Cell migration was assessed by wound healing and transwell migration assay. Matrigel invasion and xenograft model assay were performed to analyze the potential of cell invasion. Activities of Met, MEK1/2, ERK1/2, Akt, cyclinD1, caspase3 and MMP2 protein were measured by Western blot.

**Results:**

Overexpressions of MACC1 were detected in ovarian cancer tissues. Expression of MACC1 in OVCAR-3 cells was significantly down-regulated by MACC1 specific small hairpin RNA. In OVCAR-3 cells, down-regulation of MACC1 resulted in significant inhibition of cell proliferation, migration and invasion, meanwhile obvious enhancement of apoptosis. As a consequence of MACC1 knockdown, expressions of Met, p-MEK1/2, p-ERK1/2, cyclinD1 and MMP2 protein decreased, level of cleaved capase3 was increased.

**Conclusions:**

RNA interference (RNAi) against MACC1 could serve as a promising intervention strategy for gene therapy of ovarian carcinoma, and the antitumor effects of MACC1 knockdown might involve in the inhibition of HGF/Met and MEK/ERK pathways.

## Background

Ovarian cancer is one of malignant tumors in female genital system, but is the leading cause of death from gynecological cancer in the world [1]. Despite improvements in the application of aggressive cytoreductive surgery and combination chemotherapy, ovarian cancer has the most unfavorable prognosis due to its insidious onset, diagnosis at late stage, dissemination, relapse, and tendency to develop chemotherapy resistance. Though considerable efforts aim at elucidating the tumorigenesis of ovarian carcinoma, its molecular mechanism has not been completely explained.

Recently, MACC1 has been identified as a prognosis biomarker for colon cancer, which promotes proliferation, invasion and hepatocyte growth factor (HGF)-induced scattering of colon cancer cells *in vitro *and *in vivo *[2]. MET, which encodes Met protein, has been proven to be a transcriptional target of MACC1. MACC1 controls the activity and expression of MET, and regulates HGF/Met signal pathway [2]. HGF/Met pathway plays key roles in carcinogenesis, aberrant activation of Met leads to enhancement of cell proliferation, invasion and metastasis, and Met is essential for metastatic potential of many malignances [3]. Once activated by HGF, Met transmits intracellular signals and activates downstream Ras-mitogen-activated protein kinase (MAPK) and phosphoinositide 3-kinase (PI3K)/Akt pathways, which promote cell survival, migration, invasion, and suppress apoptosis [4].

MACC1 was demonstrated to be associated with poor prognosis and high risk of metastasis in colon cancer, gastric carcinoma, lung cancer, and hepatocellular carcinoma [5-8]. However, the mechanism of MACC1 implicates in ovarian cancer is still unclear. Small interfering RNA can specifically silence particular genes, and is used as a powerful tool to research gene functions and as a genetic therapy strategy for carcinoma [9]. In present study, expressions of MACC1 were detected in different ovarian tissues by immunohistochemistry, effects of MACC1 inhibition on OVCAR-3 cells were observed by RNA interference, and the possible antitumor mechanisms of MACC1 knockdown in ovarian carcinoma cells were discussed.

## Materials and methods

### Immunohistochemistry and evaluation

Paraffin-embedded 20 specimens of normal ovary, 19 specimens of benign ovarian tumor and 52 specimens of ovarian cancer tissues were obtained from Department of Pathology of Zhengzhou University. Rabbit-anti-human polyclonal MACC1 antibody (Sigma, USA) was used for immunohistochemistry assay, which was performed following the protocol of Universal SP kit (Zhongshan Goldenbridge Biotechnology, Peking, China). Positive staining of MACC1 protein presents brown in cytoplasm, partly in nucleus. Semi-quantitative counting method was used to determine positive staining described as following: Selected 10 visual fields under high power lens (× 400) randomly, counted the numbers of positive cells in 100 cells per field, calculated the average positive rate. Positive rate less than 1/3 scored as 1, more than 1/3 and less than 2/3 scored as 2, more than 2/3 scored as 3, without positive cell scored as 0. Cells without brown staining scored as 0, with mild brown staining scored as 1, with moderate brown staining scored as 2, with intense brown staining scored as 3. The final positive scores = positive rate score × staining intensity score, 0 score was negative staining (-), 1~4 scores were positive staining (+), more than 4 scores was strong positive (++).

### ShRNAs synthesis and plasmids construction

Single shRNA strands were 5'-GATCCCC-N21-TTCAAGAGA-N'21-TTTTTGGA-AA-3' (sense) and 5'-AGCTTTTCCAAAAA-N21-TCTCTTGAAN'21-GGG-3' (antisense). N21 was the sense sequence of MACC1 target oligonucleotides, N'21 was antisense sequence of MACC1 target oligonucleotides. Three different template oligonucleotides targeting MACC1 [GeneBank, NM_182762.3] were as follow: MACC1-s1, 5'-AAAGACAGAAGGAGAAAGGAA-3'; MACC1-s2, 5'-AATCAAC-

TGTCTGCTTCTAAC-3'; MACC1-s3, 5'-AATTATATGCCAGGACAGCTT-3'. As a negative control, one scrambled sequence 5'-AACAGTTATCTATGCGACAGT-3' (corresponding to MACC1-s3) was designed. These sequences were submitted to BLAST against human genome sequence to ensure that only MACC1 gene was targeted. All single shRNA strands were synthesized at Sangon Biotechnology Co., Ltd (Shanghai, China), and were annealed and ligated into the BglII and HindIII sites of linearized psuper-EGFP plasmid. The four shRNAs inserted vectors were named as psuper-EGFP-s1, psuper-EGFP-s2, psuper-EGFP-s3, and psuper-EGFP-NC respectively.

### Cell transfection

Human ovarian carcinoma OVCAR-3 cells (with high level of MACC1 expression measured in our preliminary study) were purchased from Chinese Academy of Sciences Cell Bank (Shanghai, China), and cultured in DMEM medium (HyClone, USA) supplemented with 10% fetal bovine serum (FBS), 100 U/ml penicillin and 100 mg/ml streptomycin at 37°C with 5% CO_2_. Cells were harvested in logarithmic phase of growth for all experiments described below. Cell transfection was performed following the protocol of Lipofectamine 2000 (Invitrogen, USA). The untransfected cells, empty vector (psuper-EGFP-neo) transfected cells, and nonspecific shRNA (psuper-EGFP-NC) transfected cells were used as controls. Stably transfected OVCAR-3 cells were selected with 800 μg/ml G418 (Sigma, USA) after tansfection 48 h. After 12 days, resistant colonies were trypsinized and cultured in selective medium. Names of the stably transfected cells were OVCAR-3-neo, OVCAR-3-NC, OVCAR-3-s1, OVCAR-3-s2, and OVCAR-3-s3 respectively.

### RT-PCR

Cell total RNA was isolated using Trizol Reagent (Invitrogen, USA), and first strand cDNA was synthesized from 1 μg total RNA according to the protocol of RevertAid first strand cDNA synthesis kit (Fermentas, EU). Primers used in RT-PCR were as follow: MACC1, 5'-CCTTCGTGGTAATAATGCTTCC-3' (sense) and 5'-AGGGCTTCCATTGTATTGAGGT-3' (antisense); β-actin, 5'-ACGCACC- CCAACTACAACTC-3' (sense) and 5'-TCTCCTTAATGTCACGCACGA-3' (antisense). PCR cycling parameters (19 cycles) were: denaturation (94°C, 30s), annealing (56°C, 30s) and extension (72°C, 30s). Equal amounts of PCR products were electrophoresed on 1.2% agarose gels and visualized by ethidium bromide staining. The specific bands of PCR products were analyzed by Image-Pro Plus 6.0 system, β-actin was used as a control for normalization. RT-PCR was performed for three times independently.

### Western blot

Primary antibodies used in Western blot, following manufacturer's protocols, were anti-MACC1 (Sigma, USA), anti-Met, anti-p-MEK1/2(ser212/ser218), anti-MEK1/2, anti-p-ERK1/2(Thr202/Tyr204), anti-ERK1/2 and anti-MMP2 (Santa Cruz, USA), anti-Akt, anti-p-Akt(Thr308), anti-cyclinD1, anti-cleaved caspase3 and anti-β-actin (Beyotime Biotechnology, Jiangsu, China). Total protein was extracted using Cell Lysis Buffer for Western and IP (Beyotime Biotechnology, Jiangsu, China), and protein concentration was determined using Bradford assay. Equal amounts of protein (30 μg) were separated by 10% SDS-PAGE and transferred onto PVDF membranes. The detection of hybridized protein was performed by enhanced chemiluminescence kit (Zhongshan Goldenbridge Biotechnology, Peking, China), β-actin was used as a control for normalization. The specific bands were analyzed by Image-Pro Plus 6.0 system.

### MTT assay

Planted 2 × 10^4 ^cells per well into 96-well plates, and added 100 μl medium containing 10% FBS into each well. Five duplicate wells were set up for each group. Cultured cells continuously for 7 days, added 20 μl MTT reagent (5 mg/ml, Sigma, USA) into each well, incubated for another 4 h then aspirated former medium and added 150 μl DMSO. The absorbance of sample was measured by Microplate spectrophotometer (Thermo, USA) at 492 nm. All experiments were done in triplicate. Cell growth curve was plotted versus time by origin 8 software.

### Monoplast colony formation assay

Prepared single cell suspension, seeded about 50, 100, 200 cells of each group into 6-well plates respectively. Added 2 ml medium containing 10% FBS into each well, cultured cells continuously for one week. Fixated cells with methanol for 5 min, stained cells by hematoxylin for 30 min, counted the numbers of colony (more than 10 cells per colony) under low power lens (× 100) of inverted microscope (OLYMPUS, IX71, Japan), and calculated the rate of colony formation.

### Flow cytometry analysis

About 1 × 10^6 ^cells were treated into single cell suspension with PBS solution, and were prepared following manufacture's protocol of Annexin V-FITC Apoptosis Detection Kit (Beyotime Biotechnology, Jiangsu, China). Then, rates of apoptosis were analyzed with FACScan system (BD, USA).

### TUNEL assay

Dripped single cell suspension onto microscopic slides, incubated cells for 4 h till cells were adherent. Three duplicate slides were set up for each group. Fixated cells by 4% paraformaldehyde for 30 min, blocked cells by 0.3% H_2_O_2 _for 30 min, incubated cells with 0.1% Triton X-100 for 2 min, then performed following manufacture's protocol of In situ cell death detection kit (Roche, German). Selected five visual fields under high power lens (× 400) randomly, counted the numbers of apoptotic body in 100 cells, calculated the rate of apoptosis.

### Wound healing assay

About 5 × 10^4^~1 × 10^5 ^cells were seeded into each well of 6-well plates, three duplicate wells were set up for each group, monolayer cells were obtained after cells confluence. Scratched monolayer cells with 200 μl pipette tip, washed cells 3 times with PBS, and added 2 ml medium without FBS into each well. The values of scratch were measured at 0 h and 24 h after scratching by Image Pro-Plus 6.0 system.

### Transwell migration assay

Transwell chambers (8 μm pore size; Millipore, USA) were also used to measure cell migration. Seeded 2 × 10^5 ^cells into each upper chamber with 200 μl fresh medium without FBS, added 500 μl medium with 20% FBS into each lower chamber, three duplicate wells were set up for each group. After 12 h, fixated cells with methanol for 5 min, and stained cells by hematoxylin for 30 min. Cleaned upper chamber and inverted the chamber, counted cell numbers on the lower membrane under high power lens (× 400) in five random visual fields.

### Matrigel invasion assay

Transwell chamber (8 μm pore size; Millipore, USA) covered with 100 μl of 1 mg/ml Matrigel (BD, USA) was used to measure cell invasive ability. Seeded 1 × 10^5 ^cells into each upper chamber with 200 μl fresh medium without FBS, added 500 μl medium with 20% FBS into each lower chamber, three duplicate wells were set up for each group. After 12 h, fixated cells with methanol for 5 min, and stained cells by hematoxylin for 30 min. Cleaned upper chamber and inverted the chamber, counted cell numbers on the lower membrane under high power lens (× 400) in five random visual fields.

### Xenograft model assay

The experimental protocol was approved by Zhengzhou University Ethics Committee for Animal Experimentation. Female BALB/c nu/nu mice (4-5 weeks old, 13-17 g) were purchased from Vital River Laboratory Animal Technology Co., Ltd (Peking, China), and were randomly assigned into four groups with 4 mice per group. About 1 × 10^7 ^cells were suspended in 0.2 ml PBS and injected subcutaneously into one mouse. The tumors were monitored every 5 days beginning at day 5 by measuring two perpendicular diameters with a caliper. The mice were sacrificed on the 35th day after injection, tumors were dissected and measured, and tumor volume in mm^3 ^was calculated by the formula: volume = (width)^2 ^× length/2 [10].

### Statistical analysis

Average values were expressed as mean ± standard deviation (SD). Count data were analyzed by χ^2 ^test. Measurement data were analyzed by one-way ANOVA and Bonferroni test using SPSS 17.0 software package. Difference was considered significant when *P *value was less than 0.05.

## Results

### Overexpressions of MACC1 in ovarian cancer tissues

**Figure 1:**
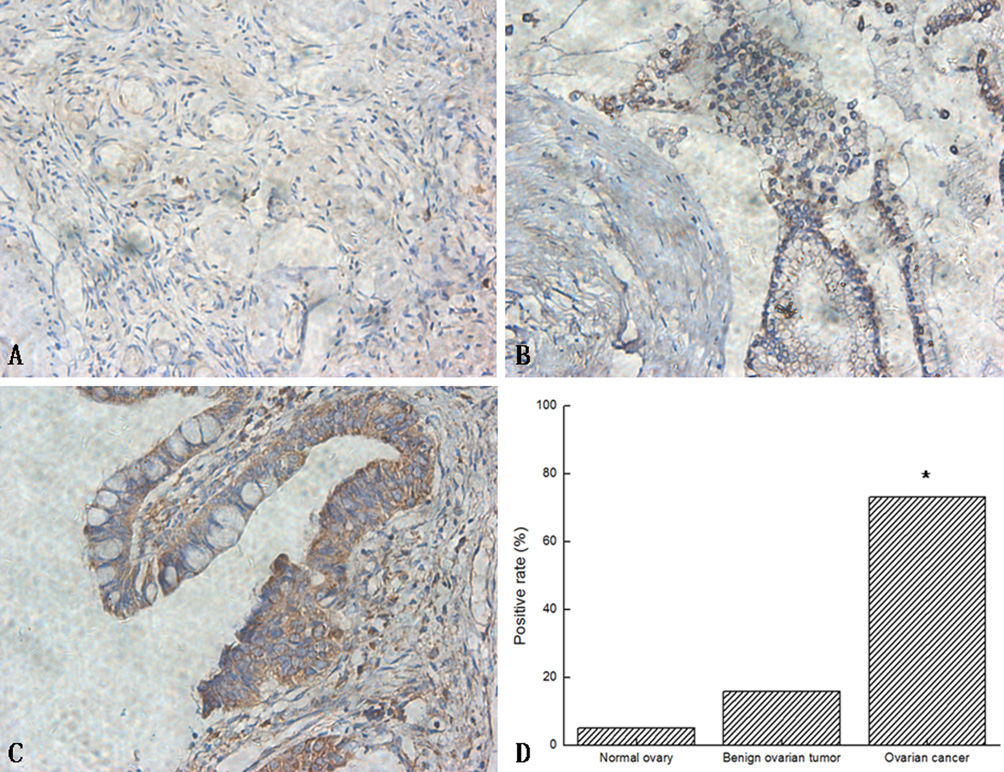
**Immunohistochemistry analysis of MACC1 expression in different ovarian tissues**. Normal ovary (A) and benign ovarian tumor (B) showed a lower staining of MACC1, but ovarian cancer (C) showed higher density staining (DAB staining, × 400). (D): Bar graphs show the positive rates of MACC1 protein. *P < 0.05 versus normal and benign ovarian tissues.

The positive rates of MACC1 in normal ovary, benign ovarian tumor and ovarian cancer tissues were detected by immunohistochemistry (Table 1). Compared to normal ovary and benign ovarian tumor, expressions of MACC1 were obviously up-regulated in ovarian cancer tissues (Figure 1), which showed abnormal expression of MACC1 might be associated with ovarian cancer.

**Table 1:** Expressions of MACC1 protein in different ovarian tissues analyzed by immunohistochemistry.

Tissue type	Variable		n	Positive n	Positive rate (%)
Normal ovarian tissue	-		20	1	5.0
Benign ovarian tumor	serous		10	2	15.8
	mucous		9	1	
	Age (years)	< 50	12	8	
		≥50	40	30	
	FIGO stage	I/II	5/11	3/5	
		III/IV	24/12	19/11	
	Histological type	Serous	30	21	
Ovarian carcinoma tissue		Mucous	22	17	
	Histological grade	G_1_	10	4	
		G_2_/G_3_	14/28	9/25	
	Ascites	No	24	16	
		Yes	28	22	
	Lymph nodes metastasis	No	32	20	
		Yes	20	18	73.1*

* χ^2 ^test. Compared with normal ovarian and benign ovarian tumor tissues *P *< 0.05.

### Down-regulation of MACC1 expressions by RNAi

**Figure 2:**
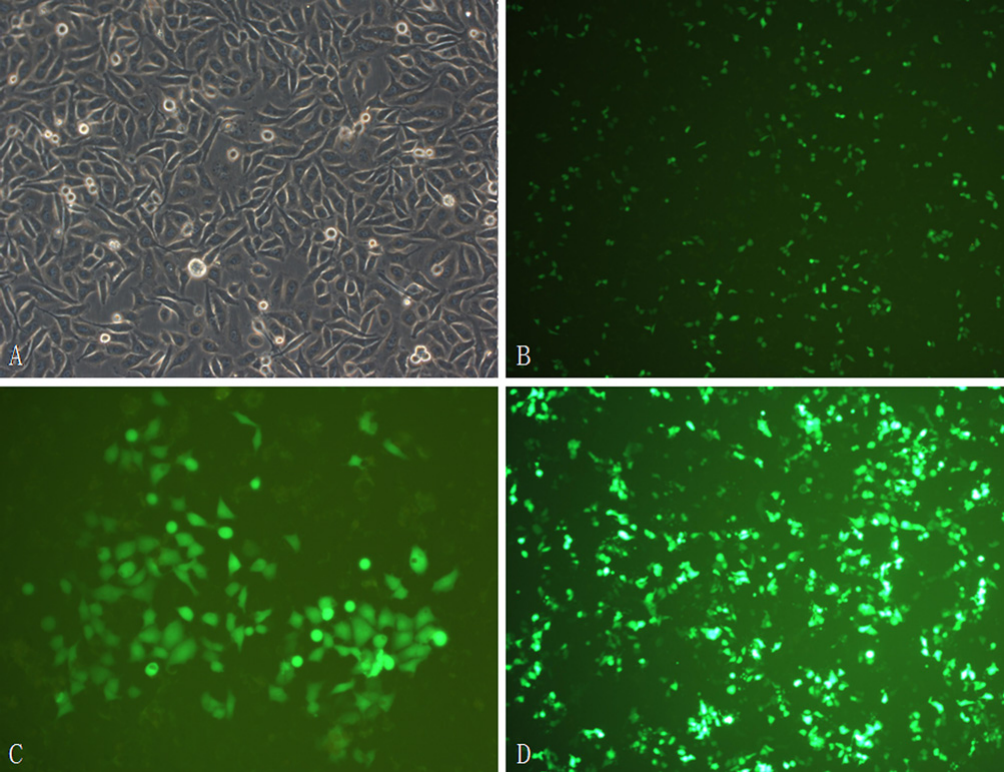
**Transfection of MACC1-shRNA into ovarian carcinoma OVCAR-3 cells**. (A): Normal OVCAR-3 cells under incandescent light (× 200). (B): After transfection 24 h, OVCAR-3-s3 cells under fluorescent light (× 100). (C): Monoplast colony of OVCAR-3-s3 cells selected by G418 for three weeks (× 200). (D): G418 resistant OVCAR-3-s3 cell line (× 100).

**Figure 3:**
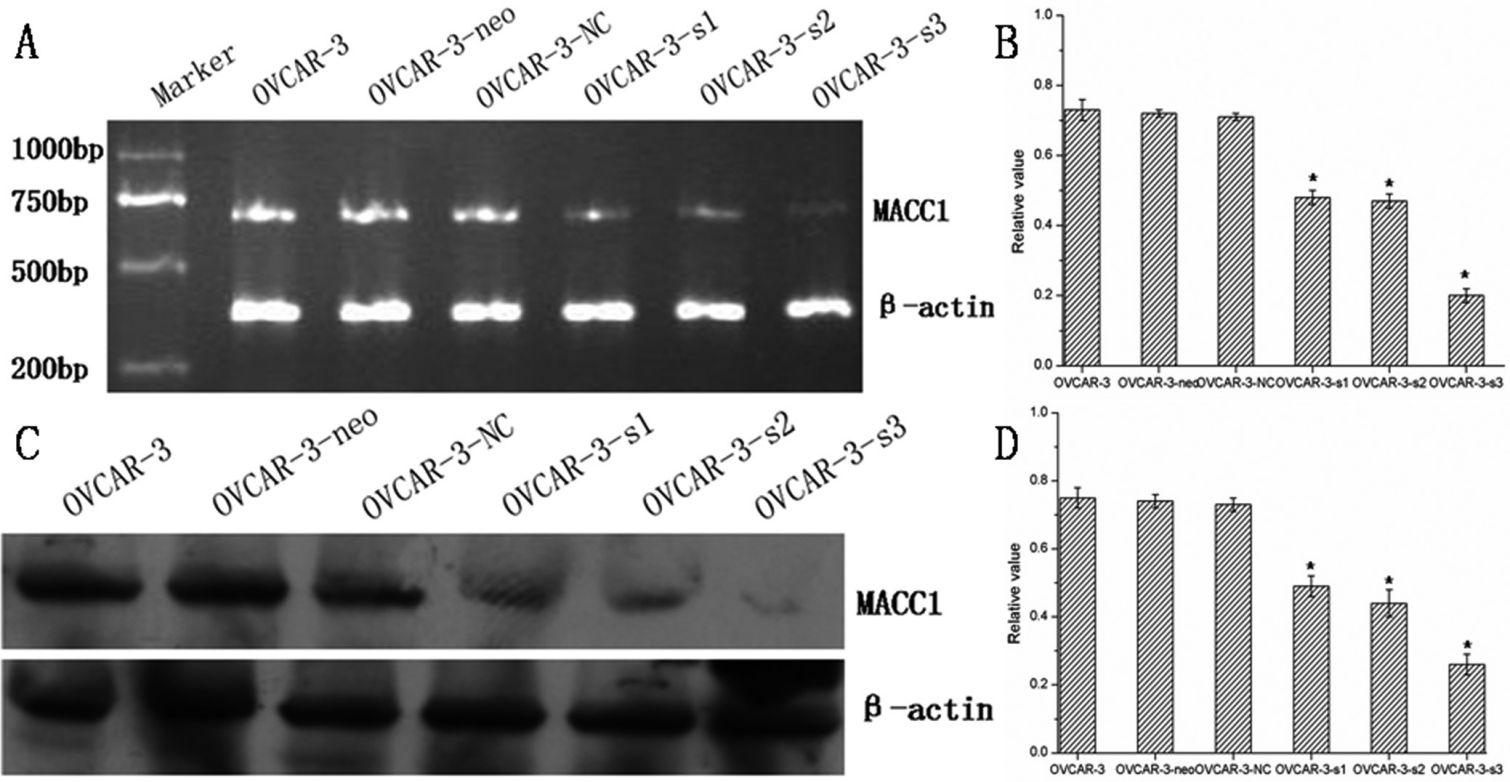
**Down-regulation of MACC1 by MACC1-shRNA in ovarian carcinoma cells**. The best inhibitory effects of MACC1 were identified in OVCAR-3-s3 cells by RT-PCR (A) and Western blot (C), which were both performed for three times independently. Bar graphs show the relative expression levels of MACC1 mRNA (B) and protein (D).*P < 0.05 versus control groups.

After transfection 48 h, transfected cells with green fluorescence under fluorescence microscopy were observed (Figure 2). Expressions of MACC1 in stably transfected cells, which were selected by G418, were measured by RT-PCR and Western blot. Compared to control cells, levels of MACC1 mRNA and protein were significantly down-regulated in OVCAR-3-s1, OVCAR-3-s2 and OVCAR-3-s3 cells, especially in OVCAR-3-s3 cells (Figure 3). According to these results, OVCAR-3-s3 cells which showed the highest inhibitory rate of MACC1 were used for further assay described below.

### Inhibition of cell proliferation and colony formation by MACC1 RNAi

**Figure 4:**
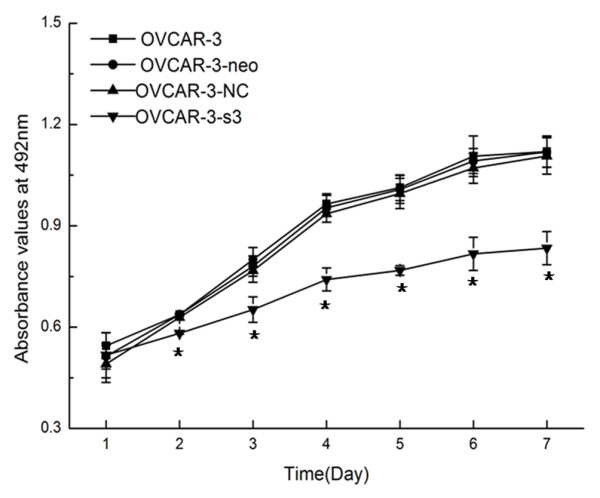
**Suppression of proliferation by MACC1 RNAi in ovarian carcinoma cells measured by MTT assay**. Obviously inhibitory effect of cell proliferation was observed from the second day after MACC1 knockdown.*P < 0.05 versus control groups.

**Figure 5:**
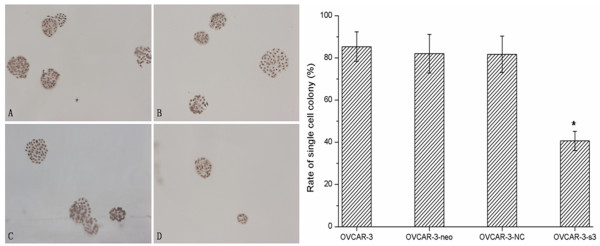
**MACC1-shRNA inhibited the monoplast colony formation of ovarian carcinoma cells**. Monoplast colony in 50-cells wells of each group. (A): OVCAR-3 cells. (B): OVCAR-3-neo cells. (C): OVCAR-3-NC cells. (D): OVCAR-3-s3 cells (Hematoxylin staining, × 100). Bar graphs show the average rates of monoplast colony formation.*P < 0.05 versus control groups.

According to Figure 4, the proliferation of OVCAR-3-s3 cells was obviously inhibited from the second day, when compared with control cells. There were no differences among OVCAR-3, OVCAR-3-neo and OVCAR-3-NC cells. In addition, OVCAR-3-s3 cells had lower rate of colony formation than control groups as shown in Figure 5. Thus, knockdown of MACC1 by RNAi could inhibit the growth of ovarian carcinoma cells.

### Apoptosis induced by MACC1 RNAi

**Figure 6:**
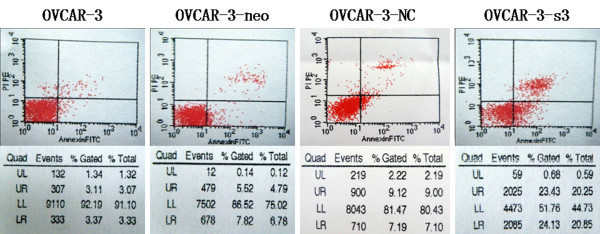
**Apoptosis induced by MACC1 RNAi in ovarian carcinoma cells**. After MACC1 inhibition, cell apoptosis was obviously induced in ovarian carcinoma cells measured by flow cytometry assay.

**Figure 7:**
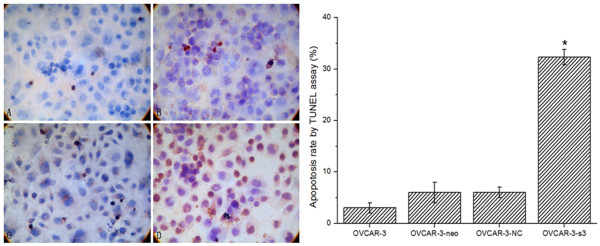
**MACC1-shRNA increased the apoptosis rate of ovarian carcinoma cells**. TUNEL assay was used to measure the apoptosis rate in OVCAR-3 cells (A), OVCAR-3-neo cells (B), OVCAR-3-NC cells (C), and OVCAR-3-s3 cells (D). DAB staining, × 400. Bar graphs show the rates of apoptosis.*P < 0.05 versus control groups.

Cell apoptosis rate measured by flow cytometer (Figure 6) in OVCAR-3-s3 cells was markedly increased to 24.13%, higher than 3.37% for OVCAR-3, 7.82% for OVCAR-3-neo, and 7.19% for OVCAR-3-NC cells (*P *< 0.05). Furthermore, TUNEL assay showed numbers of apoptosis body were increased in OVCAR-3-s3 cells (Figure 7). The results of apoptosis assay indicated the inhibitory effect of cell growth might due to the enhancement of apoptosis by MACC1 RNAi.

### Suppression of migration by MACC1 RNAi

**Figure 8:**
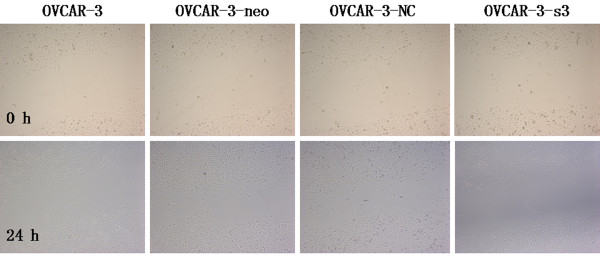
**Knockdown of MACC1 by RNAi suppressed the migration ability of ovarian carcinoma cells**. Wound healing assay was used for monolayer cell migration assay (Hematoxylin staining, × 100).

**Figure 9:**
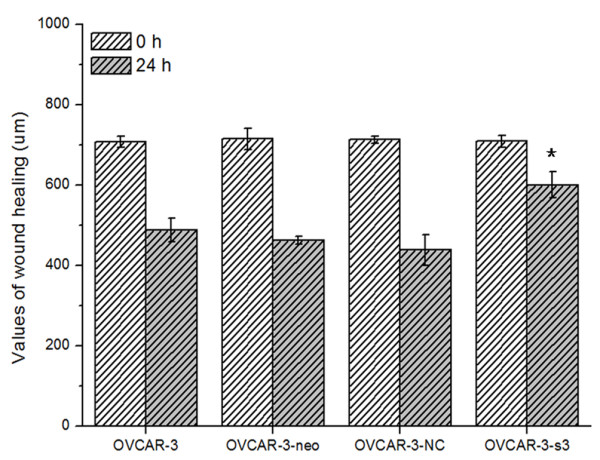
**Bar graph of the wound healing assay**. Each bar represents the value of wound healing assay. *P < 0.05 versus control groups.

**Figure 10:**
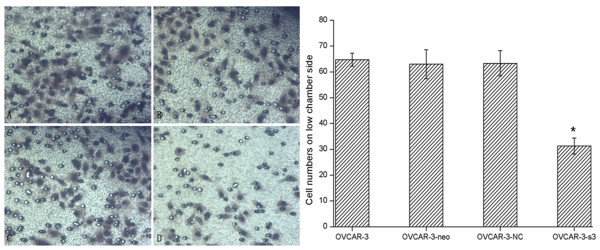
**Inhibition of MACC1 by RNAi suppressed the migration ability of ovarian carcinoma cells**. Transwell migration assay was used for cell migration ability assay. (A): OVCAR-3 cells. (B): OVCAR-3-neo cells. (C): OVCAR-3-NC cells. (D): OVCAR-3-s3 cells (Hematoxylin staining, × 400). Each bar represents the cell numbers adherent on lower membrane.*P < 0.05 versus control groups.

Compared with control groups, OVCAR-3-s3 cells showed suppressed capacity of impaired migration (Figure 8 and 9). Moreover, numbers of cell adherent on lower membranes of transwell chamber were sharply decreased in OVCAR-3-s3 group, which were shown in Figure 10. These results suggested MACC1 RNAi could suppress migration capability of ovarian carcinoma cells.

### Activity of invasion retarded by MACC1 RNAi

**Figure 11:**
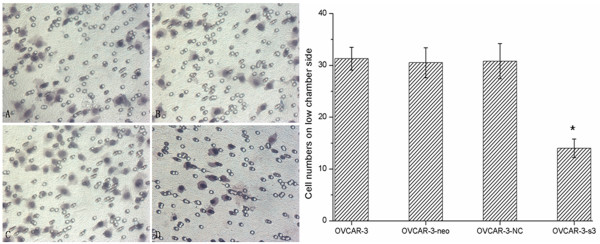
**Inhibition of invasion by MACC1 RNAi in ovarian carcinoma cells**. Cell invasive ability was assessed by Matrigel invasion assay. (A): OVCAR-3 cells. (B): OVCAR-3-neo cells. (C): OVCAR-3-NC cells. (D): OVCAR-3-s3 cells (Hematoxylin staining, × 400). Each bar represents the cell numbers adherent on lower membrane.*P < 0.05 versus control groups.

**Figure 12:**
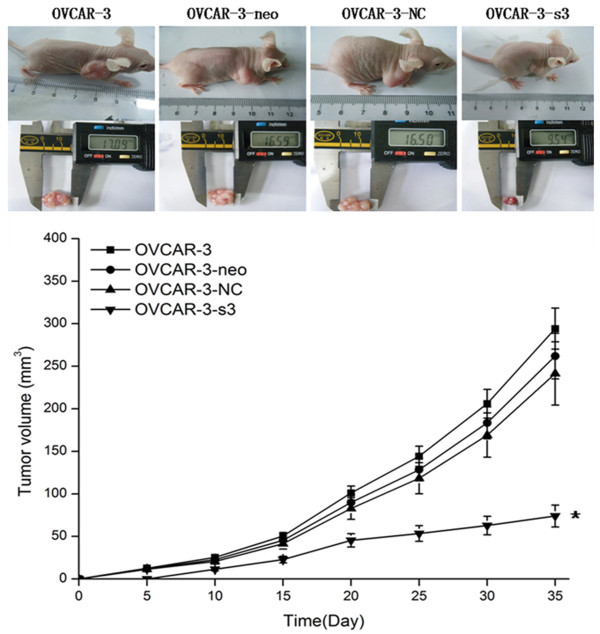
**Xenograft tumor growth of ovarian carcinoma cells was retarded by MACC1 RNAi**. On the 35th day, volumes of subcutaneous tumor in OVCAR-3-s3 group were remarkably smaller than those of control groups. Line curves represent the tumor volumes of xenograft models. *P < 0.05 versus control groups.

The numbers of cell, assessed in Matrigel invasion assay, were remarkably decreased in OVCAR-3-s3 group (Figure 11). On the other hand, the volumes of xenograft tumors removed from nude mice were retarded apparently in OVCAR-3-s3 group after 35 days. As shown in Figure 12, the growth of xenograft tumors in OVCAR-3-s3 group obviously fell behind other groups. Results of invasion assay indicated invasive potential of ovarian carcinoma cells could be retarded by MACC1 RNAi.

### Down-regulation of Met and MEK/ERK pathways activity by MACC1 RNAi

**Figure 13:**
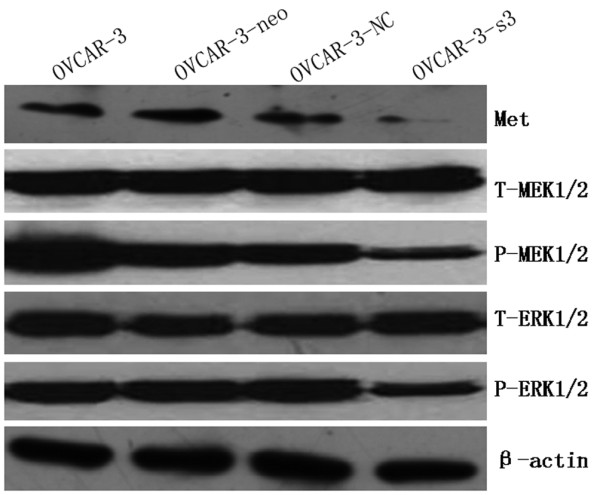
**Activities of HGF/Met and MEK/ERK signaling in ovarian carcinoma cells after MACC1 knockdown**. After MACC1 inhibition, down-regulations of Met, p-MEK1/2, p-ERK1/2 were observed in ovarian carcinoma cells analyzed by Western blot.

**Figure 14:**
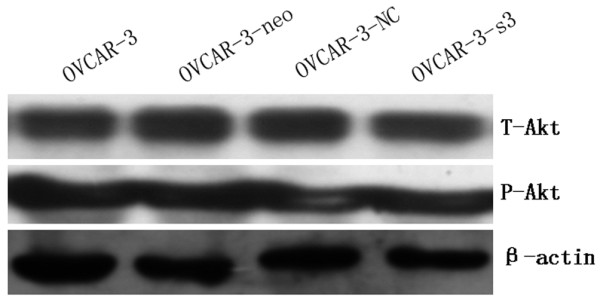
**Activity of PI3K/Akt signaling in ovarian carcinoma cells after MACC1 knockdown**. After MACC1 inhibition, none obvious changes of Akt and p-Akt expression were detected in ovarian carcinoma cells by Western blot analysis.

**Figure 15:**
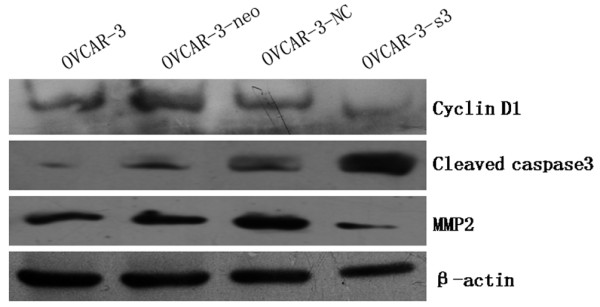
**Expressions of cyclinD1, cleaved caspase3 and MMP2 in ovarian carcinoma cells after MACC1 knockdown**. After MACC1 inhibition, expressions of cyclinD1 and MMP2 decreased, level of cleaved caspase3 was increased in ovarian carcinoma cells by Western blot analysis.

Expressions of Met, MEK1/2, p-MEK1/2, ERK1/2, p-ERK1/2, Akt and p-Akt were measured by Western blot in OVCAR-3, OVCAR-3-neo, OVCAR-3-NC and OVCAR-3-s3 cells. As a result of MACC1 knockdown, significant reductions of Met and p-MEK1/2 and p-ERK1/2 expression were observed in OVCAR-3-s3 cells. However, none obvious changes were detected on levels of total MEK1/2, total ERK1/2, total Akt and p-Akt (Figure 13 and 14). In addition, expressions of cyclinD1 and MMP2 decreased, level of cleaved caspase3 was increased after MACC1 inhibition (Figure 15).

## Discussion

Among gynecological cancers, more than 75% of ovarian carcinoma patients are suffered with advanced disease, and the majority will relapse and die of their disease [11,12]. Despite major efforts in diagnosis and improvements in the treatment of epithelial ovarian cancer, current therapies for advanced ovarian cancer are not effective enough and total survival rate of subjects with ovarian carcinoma has not changed appreciably.

MACC1 is closely associated with several types of cancer, and can serve as poor prognosis and metastatic biomarker for colon cancer, gastric carcinoma, lung cancer, and hepatocellular carcinoma [5-8]. In this study, we detected high levels of MACC1 in ovarian cancer tissues by immunohistochemistry, which showed abnormal expression of MACC1 might be associated with ovarian carcinoma. However, the relations between abnormal expression of MACC1 and ovarian carcinoma had not yet been reported.

Thus, we designed and synthesized three specific shRNAs against MACC1 gene to investigate the effects of MACC1 inhibition on ovarian carcinoma OVCAR-3 cells in present study. Results of RT-PCR and Western blot showed specific MACC1-shRNAs could effectively knockdown expression of MACC1 in OVCAR-3 cells. We also successfully obtained OVCAR-3 cell line with the best inhibitory effects of MACC1 expression for further analysis. As a consequence of MACC1 gene knockdown, the proliferation, migration and invasion of OVCAR-3 cells were obviously inhibited, but the apoptosis rate was significantly increased. These results showed inhibition of MACC1 could suppress the growth and metastatic potential of ovarian carcinoma cells *in vitro *and *in vivo*, which suggested MACC1 might implicate in the growth and metastasis of ovarian carcinoma.

MACC1 binds to a 60 bp proximal fragment of endogenous MET promoter, where contains a specific Sp1 binding site which is essential for MACC1-induced activation of MET and subsequent HGF/Met signaling consequences [13]. Once activated, Met can result in activation of several downstream signaling cascades, such as MAPK and PI3K/Akt pathways [14]. MACC1 protein contains several domains which can participate in MAPK signaling, and MACC1 can be up-regulated by MAPK pathway which has been identified to be essential for HGF-induced scattering [15-17]. In colon cancer cells, MAPK signaling could be hyperactive by transfection of MACC1, and HGF-induced cell scattering mediated by MACC1 could be abrogated by MEK specific inhibitors, whereas not by PI3K specific inhibitors [2].

After inhibition of MACC1 by RNAi in ovarian carcinoma OVCAR-3 cells, we observed that level of Met protein was down-regulated significantly, as well as expressions of p-MEK1/2 and p-ERK1/2 protein, but expression of p-Akt was uninfluenced. Therefore, we presumed that inhibition of MACC1 by RNAi might suppress the malignant behavior of ovarian carcinoma cells via HGF/Met and MEK/ERK pathways, at least in part. Furthermore, increased level of cleaved caspase3 and decreased levels of cyclinD1 and MMP2 protein were detected in ovarian carcinoma cells after RNA interference against MACC1, which suggested cyclinD1, caspase3 and MMP2 should be associated with MACC1 mediated downstream signaling.

HGF/Met signaling plays an important role in cellular growth, epithelial-mesenchymal transition, angiogenesis, cell motility, invasiveness and metastasis [18]. Deregulated HGF/C-met signaling has been observed in many tumors, including ovarian carcinoma, and been proved to contribute to tumor dissemination and metastasis [19]. MAPK and PI3K/Akt pathways have been demonstrated to implicate in cell survival, anti-apoptosis, invasion, metastasis and angiogenesis of malignancies, including ovarian carcinoma [20-22]. Because of these cascades play key roles in carcinogenesis, some specific antibodies and small molecules to neutralize or block the key regulators of these pathways have been used to inhibit tumor growth and metastasis, which exploit effective intervention strategies for malignancies [19,23,24]. According to previous reports and the results described above, we considered that MACC1, as a key regulator and upstream signaling of these pathways, could be a potential therapeutic target for ovarian cancer.

## Conclusions

In summary, our data showed that MACC1 might implicate in growth and metastasis of ovarian carcinoma. In ovarian carcinoma cells, the antitumor effects of MACC1 RNAi might involve in the inhibition of HGF/Met and MEK/ERK pathways. As a key regulator of HGF/Met signaling, RNA interference against MACC1 could serve as a promising intervention strategy for gene therapy of ovarian carcinoma.

## Abbreviations

ERK: extracellular signal-regulated kinase; HGF: hepatocyte growth factor; MACC1: metastasis-associated in colon cancer 1; MAPK: mitogen-activated protein kinase; MEK: mitogen-activated protein kinase kinase; Met: hepatocyte growth factor receptor; PI3K: phosphoinositide 3-kinase; RNAi: RNA interference; shRNA: small hairpin RNA.

## Competing interests

The authors declare that they have no competing interests.

## Authors' contributions

ZR participated in design of the study, carried out molecular genetic studies, drafted manuscript and performed statistical analysis. SH participated in design of the study and reviewed manuscript. CZ, RF and HH carried out immunohistochemistry and participated in statistical analysis. WQ participated in design of the study and helped to draft manuscript. All authors read and approved the final manuscript.
